# Uncovering hidden genetic variations: long-read sequencing reveals new insights into tuberous sclerosis complex

**DOI:** 10.3389/fcell.2024.1415258

**Published:** 2024-07-31

**Authors:** Jing Duan, Shirang Pan, Yuanzhen Ye, Zhanqi Hu, Li Chen, Dachao Liang, Tao Fu, Lintao Zhan, Zhuo Li, Jianxiang Liao, Xia Zhao

**Affiliations:** ^1^ Department of Neurology, Shenzhen Children’s Hospital, Shenzhen, Guangdong, China; ^2^ Grandomics Biosciences, Beijing, China; ^3^ Shenzhen A-Smart Medical Research Center, Shenzhen Research Institute of the Chinese University of Hong Kong, Shenzhen, Guangdong, China

**Keywords:** mosaic variants, structural variation, Alu element, SINE-VNTR-Alus, optical genome mapping, mosaic complex structural variant

## Abstract

**Background:**

Tuberous sclerosis is a multi-system disorder caused by mutations in either *TSC1* or *TSC2*. The majority of affected patients (85%–90%) have heterozygous variants, and a smaller number (around 5%) have mosaic variants. Despite using various techniques, some patients still have “no mutation identified” (NMI).

**Methods:**

We hypothesized that the causal variants of patients with NMI may be structural variants or deep intronic variants. To investigate this, we sequenced the DNA of 26 tuberous sclerosis patients with NMI using targeted long-read sequencing.

**Results:**

We identified likely pathogenic/pathogenic variants in 13 of the cases, of which 6 were large deletions, four were InDels, two were deep intronic variants, one had retrotransposon insertion in either *TSC1* or *TSC2*, and one was complex rearrangement. Furthermore, there was a *de novo* Alu element insertion with a high suspicion of pathogenicity that was classified as a variant of unknown significance.

**Conclusion:**

Our findings expand the current knowledge of known pathogenic variants related to tuberous sclerosis, particularly uncovering mosaic complex structural variations and retrotransposon insertions that have not been previously reported in tuberous sclerosis. Our findings suggest a higher prevalence of mosaicism among tuberous sclerosis patients than previously recognized. Our results indicate that long-read sequencing is a valuable approach for tuberous sclerosis cases with no mutation identified (NMI).

## Introduction

Tuberous sclerosis is a genetic disorder that affects multiple organs and has an estimated incidence of 1 in 10,000 to 1 in 6,000 newborns (Northrup et al., 1993; Northrup et al., 2021). It is characterized by the presence of benign tumors in multiple organs, such as the brain, kidneys, skin, heart, lungs, and eyes, and is associated with a range of neuropsychiatric symptoms like epilepsy, intellectual disability, and autism spectrum disorder (Northrup et al., 1993; Ekong et al., 2016). If left untreated, central nervous system tumors and renal disease can lead to significant morbidity and mortality, and therefore, early diagnosis and regular monitoring and treatment are essential (Northrup et al., 2021).

In 2012 and 2021, the clinical diagnostic criteria were developed and updated for diagnosing individuals with suspected tuberous sclerosis (Davis et al., 2017; Northrup et al., 2021). These criteria enable the classification of patients into “definite TSC” or “possible TSC” depending on the number of major and minor clinical features they exhibit. Notably, the clinical manifestations of tuberous sclerosis can significantly vary between and within families and may evolve over time and at different ages (Davis et al., 2017; Kingswood et al., 2017).

Molecular diagnosis of tuberous sclerosis is established through the identification of a pathogenic variant of *TSC1* or *TSC2*, regardless of the clinical findings (Krueger et al., 2013; Northrup et al., 2021). As technology has advanced, various techniques have been used for genetic testing of tuberous sclerosis, such as Sanger sequencing (Au et al., 2007), single-strand conformational analysis (Au et al., 2007), denaturing high-performance liquid chromatography (Dabora et al., 2001), long-range PCR (Dabora et al., 2001), multiplex ligation-dependent probe analysis (MLPA), targeted next-generation sequencing (NGS) (Nellist et al., 2015), deep sequencing (Ye et al., 2022; Blasco-Perez et al., 2023; Klonowska et al., 2023), and whole-exome sequencing and genome sequencing (Ogorek et al., 2020). Generally, a vast majority of patients (85%–90%) are identified as carrying a heterozygous germline pathogenic variant in either *TSC1* or *TSC2* (Dabora et al., 2001; Peron et al., 2018). In addition, the majority of the remaining patients may be identified as having mosaic variants via deep sequencing (Qin et al., 2010; Manzanilla-Romero et al., 2021; Blasco-Perez et al., 2023). However, few patients have “no mutation identified” (NMI) results even after using various techniques. Our hypothesis is that the causal variant in these patients with NMI may be a structural variant or a deep intronic variant, which cannot be identified by the standard, NGS-based techniques.

In recent years, various approaches have been developed to enhance the ability to detect structural variation, such as long-read sequencing and optical genome mapping (OGM) (Shieh et al., 2021). The long-read sequencing technology can generate reads that span from 1,000 to >1 million base pairs (bp) (Logsdon et al., 2020; Mastrorosa et al., 2023), resulting in better detection of structural variations than possible with NGS, the traditional short-read sequencing technology. In 2023, Ura et al. reported a novel intron retention in a patient with tuberous sclerosis complex using cDNA long-read sequencing (Ura et al., 2023). Therefore, long-read sequencing is considered an effective way to discover unknown pathogenic variants in Mendelian diseases (Merker et al., 2018).

In this work, we conducted genetic analyses of full *TSC1* and *TSC2* in 26 patients with NMI having definite TSC using a targeted long-read sequencing approach based on the PacBio SMRT platform (Pacific Biosciences of California Inc., Menlo Park, CA, United States) to identify missed pathogenic variants.

## Materials and methods

### Patients

The Ethics Committee of Shenzhen Children’s hospital authorized the study, and 26 patients having tuberous sclerosis with NMI in either *TSC1* or *TSC2* were enrolled between 2022 and 2023. The inclusion criteria were as follows: a definite diagnosis of tuberous sclerosis according to the 2021 Updated International tuberous sclerosis Diagnostic Criteria and Surveillance and Management Recommendations (Northrup et al., 2021), no mutation identified by clinical genetic testing (whole-exome sequencing, gene panel sequencing, deep sequencing, and MLPA), and no pathogenic variants identified in *TSC1* or *TSC2* upon reanalysis of the patient’s clinical genetic testing data from NGS, if available. The exclusion criteria were as follows: a history of bone marrow stem cell transplantation and a history of blood transfusion within the past year. After obtaining written informed consent from the parents, peripheral blood samples were collected from all patients and also from the parents, if possible. Notably, in the TSC-T28 case, the skin tissue of the fetus was collected after induction of labor.

### Targeted long-read sequencing

Genomic DNA was extracted from peripheral blood samples and the skin using the Blood Genome DNA Extraction Kit (Sangon Bioengineer Co., Shanghai, China) and HiPure Tissue & Blood DNA Kit (Magen, Guangzhou, China), respectively, according to the manufacturers’ instructions. Then, the quantity and quality of the extracted DNA were then evaluated using Qubit 3.0 (Thermo Fisher Scientific Inc., Carlsbad, CA, United States) and agarose gel electrophoresis, respectively. Subsequently, 3 μg of genomic DNA was sheared into 1–6 kb fragments using a G-Tube (#520079, Covaris) centrifugation (1,500 *× g*, 2 min, twice). The fragments were then purified and end-repaired, followed by A-tailing at their 3′termini and adapter ligation through pre-capture amplification. Targeted sequence capture was done by pooling indexed PCR products and hybridization with custom capture probes. DNA probes of 120 bases were designed to cover *TSC1*, *TSC2*, and other 26 genes (Supplementary Material) and synthesized, including all the exons, introns, 5′-UTRs, and 3′-UTRs. The probes corresponding to repetitive sequences in the human genome were excluded. The purified DNA fragments were amplified by PCR and quantified, and then, they were subjected to sequencing on the long-read sequencing platform PacBio Sequel II (Pacific Biosciences) as per the manufacturer’s standard conditions. After data collection, the raw sequencing reads were demultiplexed according to their corresponding barcodes using PacBio’s Lima tool (version 1.11.0, Pacific Biosciences). Next, circular consensus sequencing (CCS) subreads were processed to generate CCS reads using the CCS software (Pacific Biosciences, version 6.4.0). Subsequently, the CCS reads were mapped on a reference genome (GRCh37, hg19) using minimap2 (version 2.17). Finally, structural variants (SVs) and single nucleotide variants (SNVs) of each sample were detected using Sniffles (version 1.0.12) and DeepVariant (version 0.10.0), respectively. Variant nomenclature was based on RefSeq NM_000368.5 for *TSC1* and RefSeq NM_000548.5 for *TSC2*.

### Long-read whole-genome sequencing by Oxford Nanopore technology

Genomic DNA of TSC-T5 was sequenced using the PromethION sequencer [Oxford Nanopore Technologies (ONT), Oxford, United Kingdom] to determine the structure of the SV. Library preparation was conducted using a 1D Genomic DNA ligation kit (SQK-LSK110) as per the manufacturer’s instructions. Herein, 5 µg of genomic DNA was size-selected with a Blue Pippin System (Sage Science, Beverly, MA) to eliminate small DNA fragments. Then, end-repair and dA-tailing of DNA fragments was performed using the Ultra II End Prep module (E7546L, New England Biolabs, Ipswich, MA, United States). The purified dA-tailed sample, the Blunt/TA ligase master mix (#M0367, New England Biolabs), and the tethered 1D adapter mix (SQK-LSK110, ONT) were combined and purified. The library was sequenced on R9.4 flow cells using PromethION sequencers. PromethION database calling was performed with Guppy v5.0.16 (ONT), and only pass reads (qscore ≥ 7) were used for further analysis. ONT long reads were aligned with the human reference genome (GRCh37/hg19) using Minimap2 (version 2.17). Finally, SVs and SNVs of each sample were detected using Sniffles (version 1.0.12) and DeepVariant (0.10.0), respectively.

### Optical genome mapping

Ultra-high-molecular-weight (UHMW) gDNA was isolated from peripheral blood using the SP Blood and Cell Culture DNA Isolation Kit from Bionano Genomics (San Diego, CA, United States) and following the Bionano Prep^®^ SP Frozen Human Blood DNA Isolation Protocol v2 (Document Number: 30,395). Subsequently, the isolated UHMW gDNA was fluorescently tagged using the Bionano Prep DLS Kit (Bionano Genomics, San Diego, CA, United States) according to the Bionano Prep Direct Label and Stain (DLS) Protocol (Document Number: 30,206). The labeled UHMW gDNA was then loaded onto a Saphyr chip and linearized and imaged on the Saphyr instrument (Bionano, San Diego, United States) with an aim to attain a throughput of 2T for the identification of mosaic SVs. Subsequently, the data were processed in the Bionano Solve software version 3.7 using the *de novo* assembly, variant annotation pipeline and Rare Variant pipeline.

### Droplet digital PCR

Genomic DNA was extracted from peripheral blood using the Magnetic Blood Genomic DNA Kit (Tiangen Biotech, Beijing, China). Then, digital PCR (ddPCR) was performed using the QX200 AutoDG Droplet Digital PCR system (Bio-Rad, United States) according to a previously established protocol (Liu et al., 2020). To validate the mosaic deletion of exon 17–22 in TSC-T16, two pairs of primers were designed on exon 19 and exon 21, and one primer on the *RPP30* gene was used as an internal amplification control. (Supplementary Table S1). For ddPCR amplification, we used the original DNA and its 2× diluted version and calculated the variant allele fraction (VAF) by taking the difference between the amplified product concentration of exon 19 and exon 21 from the amplified product concentration of *RPP30* and dividing this difference by the amplified concentration of RPP30.

### Multiplex ligation-dependent probe analysis

Multiplex ligation-dependent probe analysis (MLPA) was used in cases wherein long-read sequencing data indicated the presence of an SV involving exon regions and the clinical genetic test results of the patient did not include an MLPA test result. The SALSA MLPA P064 *TSC2* kit and SALSA MLPA P124-C3 *TSC1* kit (MRC-Holland, Amsterdam, Netherlands) were used, and the results were analyzed by Coffalyser.Net™ (MRC-Holland).

### Sanger validation of candidate variants

Standard Sanger sequencing was performed to validate SNVs and InDels. For germline variants, when parental samples were available, Sanger sequencing was used to verify the variant source in both parents.

To verify SVs, Primer6 software (Primer-E Ltd., Plymouth, United Kingdom) was used to pick primers for the breakpoint junction sequences of both 5′and 3′ends of SVs. PCR was performed as per the manufacturer’s protocols, and the PCR products were then analyzed by agarose gel electrophoresis. Sanger sequencing was used to sequence the breakpoint junction products, and the precise breakpoint positions were identified by mapping the Sanger sequencing sequences to the hg19 reference genome using the BLAST-like alignment tool (Kent, 2002).

### Multiplex short tandem repeats (STRs) analysis

The biological relationship between parents and the patient was confirmed using 12 microsatellite loci and a sex determination locus of amelogenin (AMEL) gene, which was then analyzed by PCR and capillary electrophoresis (primers in Supplementary Table S4). All PCRs were performed with Biorad T100 thermal cycler using standard cycle parameters: 3 min at 94°C, followed by 35 cycles of 94°C for 30 s, 55°C for 30 s, and 72°C for 45 s, and finally 72°C for 5 min. Electrophoresis and visualization of alleles were performed on an ABI 3730 DNA analyzer (Applied Biosystems, Carlsbad, CA, United States). Allele sizes were estimated using GeneMarker version 2.2.0 software (SoftGenetics, PA, United States).

## Results

### Patients and summary of the findings

We performed long-read sequencing for 26 patients with tuberous sclerosis to identify any missed pathogenic variants and evaluate the efficacy of long-read sequencing. *TSC1* and *TSC2* were covered comprehensively in the 26 patients, with an average depth of 133X for *TSC1* and 68X for *TSC2*. The average sequence read length was 3,598 bp.

**FIGURE 1:**
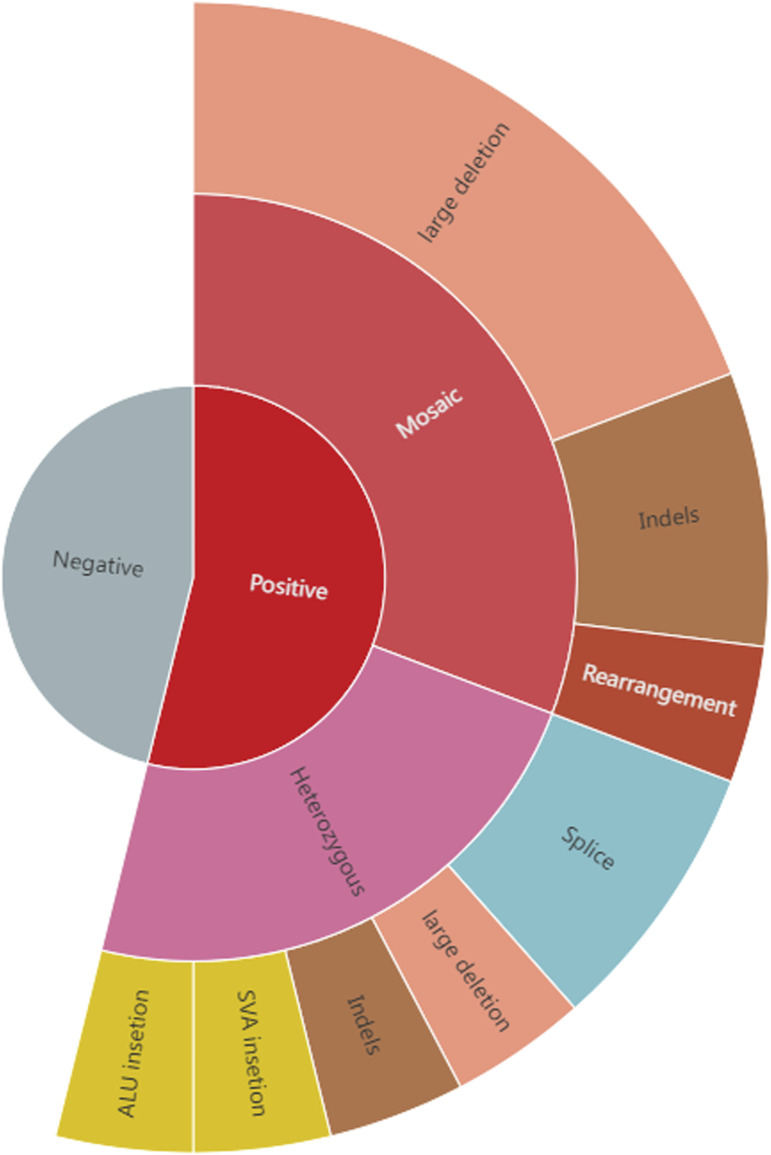
The positive rate and variant types in 26 tuberous sclerosis NMI patients. Long-read sequencing of the 26 patients with NMI uncovered variants in 14 (53.8%) of them, with 8 being mosaic and 6 being germline. These mosaic variants included 5 large deletions, 2 Indels and 1 complex structural variant, while the germline variants comprised 1 large deletion, 1 InDel, 2 splice variants and 2 retrotransposon insertions (1 ALU insertion and 1 SVA insertion).

None of the patients had a family history of tuberous sclerosis. Furthermore, none of them had no pathogenic variants identified in previous clinical genetic testing, with details of each patient’s genetic testing summarized in Table 1. Fourteen (14/26, 53.8%) of them underwent deep sequencing, whereas the remaining cases underwent routine clinical NGS sequencing of *TSC1* and *TSC2*. Long-read sequencing of the 26 patients with NMI revealed variants with a high suspicion of pathogenicity in 14 (53.8%) patients, including 8 SVs, 4 InDels, and two splice variants; eight of these variants were mosaic and six were germline (Table 1; Figure 1). There were two retrotransposon insertions as well; however, they were not picked up during clinical testing. Out of these 14 variants, 13 were identified as likely pathogenic or pathogenic, with one being identified as a variant of unknown significance. The specific details of the variant ACMG classifications are available in Supplementary Table S2. Our analysis specifically targeted the genes linked to tuberous sclerosis complex, as well as all genes sequenced by long-read sequencing. We did not identify any clinically significant unexpected variants in any other gene.

**TABLE 1:** Variants identified in the tuberous sclerosis NMI subjects.

Patient	Gene	Variant	Variation type	Source of variation	Mutant allele frequency (%)	ACMG Pathogenicity	Previous clinical genetic test	Validation methods
Germline variants								
TSC-T3	*TSC2*	c.848+281C>T	Splice	*de novo* ^a^	50	Pathogenic	Deep sequencing, WES	Sanger
TSC-T4	*TSC2*	c.848+281C>T	Splice	Not maternal	50	Pathogenic	Deep sequencing	Sanger
TSC-T20	*TSC2*	c.4006–18_4006-17insAlu4006-17_4006-4dup	InDel	*de novo* ^b^	50	VUS	Deep sequencing, NGS panel	Sanger
TSC-T11	*TSC2*	c.4937_4960dup	InDel	*de novo* ^a^	50	Likely pathogenic	NGS panel	Sanger
TSC-T25	*TSC2*	NC_000016.9:2091899_2130541del	SV	NA	50	Pathogenic	NGS panel	Sanger, MLPA
TSC-T27	*TSC2*	NM_000548.3:c.1271_1272insSVA1272_1285dup	SV	*de novo* ^a^	50	Likely Pathogenic	Deep sequencing	Sanger, OGM
Mosaic variants								
TSC-T5	*TSC2*	NC_000016.9:g.2117813_2117814ins [GG; 2086537_2115686inv; GCTTGCAGGTGCGCAT; 2115777_2127813dup]	SV	—	8.15	Pathogenic	Deep sequencing MLPA	Sanger, OGM, ONT-WGS
TSC-T17	*TSC2*	c.4715_4727delinsTGGCTCCTACAGGTACAGGTACAGAT NP_000539.2:p.(T1572Mfs*35)	InDel	—	9.68	Pathogenic	NGS panel	Sanger
TSC-T7	*TSC1*	NC_000016.9:g.135782986_135789396del	SV	—	2.06	Pathogenic	Deep sequencing MLPA	Sanger
TSC-T12	*TSC2*	c.2085_2546-384del NC_000016.9:2121923_2125416del	SV	—	8.93	Pathogenic	NGS panel	Sanger, MLPA
TSC-T15	*TSC2*	NC_000016.9:2093996_2127706del	SV	—	14.29	Pathogenic	WES	Sanger, MLPA, OGM
TSC-T16	*TSC2*	NC_000016.9:2118894_2125546del	SV	—	8.51	Pathogenic	Trio-WES	ddPCR, MLPA
TSC-T23	*TSC2*	NC_000016.9:2127219_2142640del	SV	—	15.24	Pathogenic	Deep sequencing (blood + brain tissue)	Sanger, MLPA
TSC-T28	*TSC2*	NM_000548.3:c.1130_1258-163del	InDel	—	6.02	Pathogenic	Deep sequencing, WES, WGS	Sanger

^a^ : *de novo with confirmed parental relationships*.

^b^ : *de novo with unconfirmed parental relationships*.

ACMG, american college of medical genetics guideline; NGS, next-generation sequencing; OGM, optical genome mapping; ddPCR, droplet digital PCR; MLPA, multiplex ligation-dependent probe analysis; SV, structural variant; InDels, insertion–deletion variants; VUS, variant of uncertain significance; ONT-WGS, Long-read whole-genome sequencing by Oxford Nanopore technology; NA, unavailable.

### Large deletions

**FIGURE 2:**
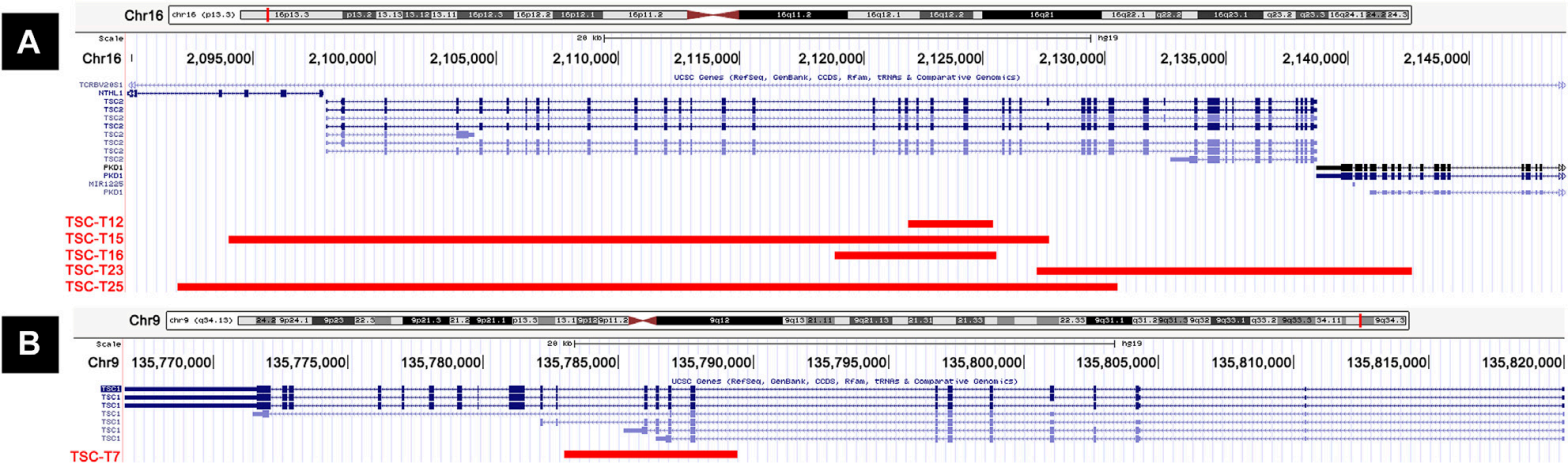
Cases with large deletions in our series. **(A)** Five patients (TSC-T12, TSC-T15, TSC-T16, TSC-T23, and TSC-T25) have large deletions on *TSC2* gene. **(B)** TSC-T7 has a large deletion on TSC1 gene.

In this cohort of patients with NMI, the most common pathogenic variant was large deletions, which was found on *TSC1* (TSC-T7) in one sample and on *TSC2* (TSC-T12, TSC-T15, TSC-T16, TSC-T23, and TSC-T25) in five samples (Figure 2). Among the latter, the 38.6-kb deletion in TSC-T25 was observed to be heterozygous, while the other four were mosaicism. The size of the mosaic deletions varied from 3.5 kb to 38.6 kb and the VAF ranged from 2.1% to 15.2%. PCR and Sanger sequencing were used to determine the breakpoint junctions of all the deletions (Supplementary Figures S1–S6), except in TSC-T16, wherein the deletion’s breakpoints were located within repetitive elements, making it difficult to amplify the breakpoint junctions. Therefore, droplet digital PCR (ddPCR) was used to accurately measure the somatic mosaicism of the deletion, with an average VAF of 9.15% being indicated by ddPCR. Moreover, the deletion variant in TSC-T15 was further validated by OGM, which revealed a 32-kb deletion overlap with *TSC2* (Supplementary Figure S1C). Nonetheless, due to a homozygous insertion variant of ∼2.3 kb at the chr16:2154992 position (PKD1 gene), the size and location of the deletion reported by OGM were slightly smaller and nearer to *PKD1* than indicated by long-read sequencing results.

The large deletions in TSC-T7, TSC-T12, and TSC-T16 only impacted either *TSC1* or *TSC2*, particularly the deletion of exon 9–12 of *TSC1* and exon 19–22 or exon 17–22 of *TSC2*. Moreover, the deletions of TSC-T15, TSC-T23, and TSC-T25 affected not only *TSC2* but also other genes nearby; the TSC-T23 variant encompassed exon 39–46 of *PKD1*, whereas TSC-T15 and TSC-T23 variants spanned exon 1–3 and exon 1–4 of *NTHL1*, respectively. *NTHL1* encodes a DNA N-glycosylase belonging to the endonuclease III family. Biallelic *NTHL1* pathogenic variants are associated with Familial adenomatous polyposis 3, an autosomal recessive inherited disorder (Weren et al., 2015).

The *TSC1/TSC2* MLPA assays for TSC-T25 revealed a heterozygous deletion of exon 1–30 in *TSC2*, which was consistent with the long-read sequencing results. Conversely, the *TSC1/TSC2* MLPA assays of TSC-T7, TSC-T12, and TSC-T16 yielded negative results, as the VAFs of these deletions were 2.1% 8.9%, and 8.5%, respectively, which were below the detectable range of MLPA. Conversely, the VAFs of TSC-T15 and TSC-T23 were 14.3% and 15.2% respectively, and the MLPA data of these two patients indicated that the final ratio of probes was slightly lower in the missing region than in the normal region (Supplementary Figure S1B; Supplementary Figure S5) but still within the normal range (0.80 < final ratio < 1.20).

### Mosaic complex rearrangement

The mosaic complex rearrangement was identified in TSC-T5, who was a 12-year-old girl. At the age of 10 years, she was admitted to our hospital after a routine physical examination revealed hamartoma in both kidneys. Further examination revealed facial angiofibroma, a fibrous cephalic plaque, renal angiomyolipomas, liver hamartoma, multiple cortical tubers, and a subependymal nodule, leading to a definite diagnosis of tuberous sclerosis. Subsequent deep sequencing and MLPA of *TSC1* and *TSC2* did not reveal any pathogenic variants.

**FIGURE 3:**
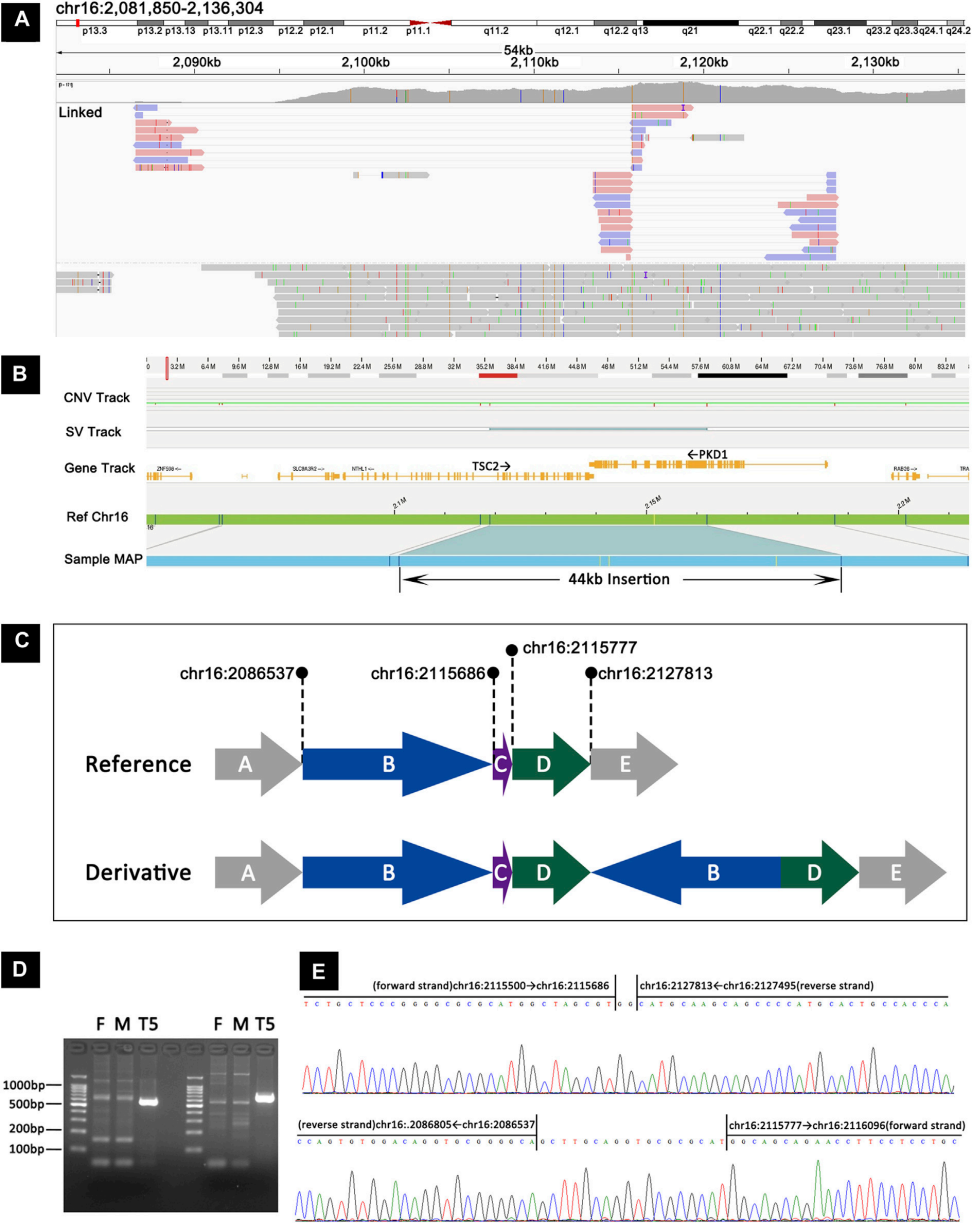
Detection and validation of the mosaic complex rearrangement in TSC-T5 by targeted long-read sequencing, long-read whole-genome sequencing, OGM, and Sanger sequencing. **(A)** Targeted long–read sequencing revealed the junction between chr16:2083537 (forward strand) and chr16:2115777 (reverse strand) and between chr16:2115686 (forward strand) and chr16:2127813 (reverse strand). **(B)** OGM showing a mosaic 44-kb insertion in *TSC2*. **(C)** A schematic representation of the mosaic complex rearrangement. **(D)** PCR products from junction 1 and junction 2 were analyzed using agarose gel electrophoresis, and the junction sequences were found to be present in TSC-T5 but not in her parents. **(E)** Sanger sequencing corroborated the two junctions proposed by long-read sequencing, revealing an extra 2-bp sequence in junction 1 and 18-bp sequence in junction 2.

Using targeted long-read sequencing, we were able to pinpoint two breakpoint junction sequences. These comprised the junction between chr16:2083537 (forward strand) and chr16:2115777 (reverse strand) and the junction between chr16:2115686 (forward strand) and chr16:2127813 (reverse strand; Figure 3A). To gain further insight, we used long-read whole-genome sequencing with Oxford Nanopore technology, which has a read length of ∼50 kb and provides more information about the breakpoint junction sequences than targeted long-read sequencing. Additionally, we applied the optical genome mapping (OGM) to this sample, which revealed a mosaic 44-kb insertion in *TSC2* (Figure 3B). Integration of data from all three platforms enabled precise characterization of this variant and facilitated the generation of a schematic representation (Figure 3C). The representation illustrates a duplication event involving segments B (chr16:2086537-2115686, which include the last four exons of *SLC9A3R2*, *NTHL1*, and exons 1–15 of *TSC2*) and D (chr:2115777-2127813 encompassing exons 16–24 of *TSC2*), wherein the reverse orientation of segment B is connected to the forward orientation of segment D, ultimately resulting in an insertion at the chr16:2127813 locus (intron 26 of *TSC2*).

To validate the two breakpoint junctions, primers were designed to amplify the junction sequences. The glue diagram showed that the junction sequences were present in the patient sample but not in the parent sample (Figure 3D). Sanger sequencing results confirmed the two junctions proposed by long-read sequencing and also revealed that an extra 18-bp sequence was inserted in the junction of chr16:2083537 and chr16:2115777 and that a 2-bp sequence was inserted in the junction of chr16:2115686 and chr16:2127813 (Figure 3E).

### Retrotransposon insertion

**FIGURE 4:**
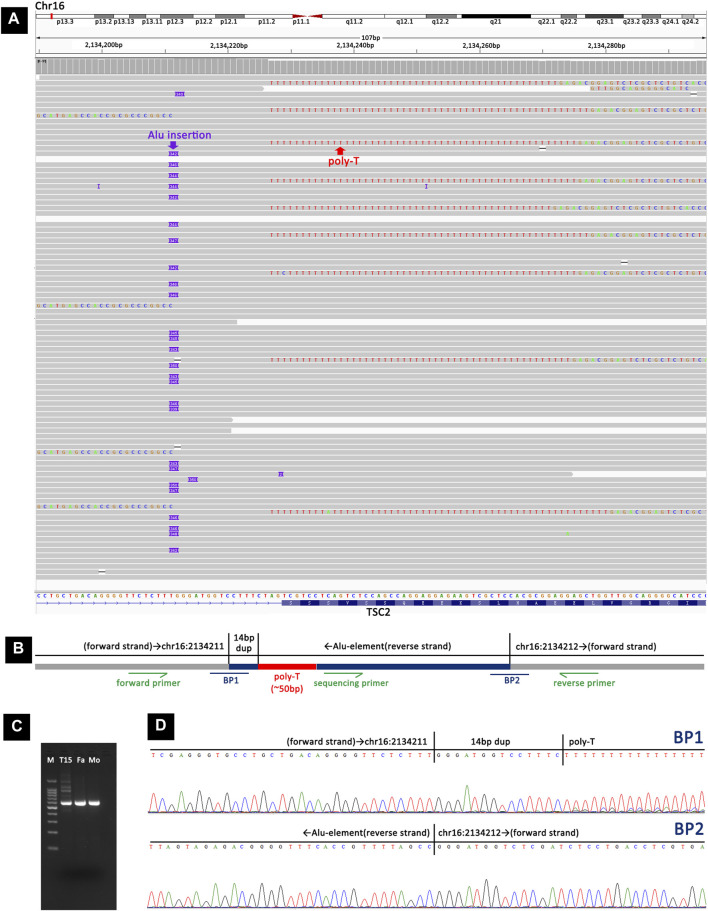
Detection and validation of the Alu element insertion in TSC-T20 via long-read sequencing and Sanger sequencing. **(A)** Long-read sequencing revealed a heterozygous Alu element insertion, c.4006-18_4006-17insAlu4006-17_4006-4dup, within intron 31 of *TSC2*. **(B)** A schematic representation of the Alu element insertion. BP1 and BP2 represent the two breakpoint sequences. Primers were designed adjacent to the inserted fragment, and an additional sequencing primer was used to sequence the two breakpoints. **(C)** Agarose gel electrophoresis of PCR products showing that the insertion was present in TSC-T20 but not in her parents. **(D)** Sanger sequencing of the PCR products from TSC-T20 validated the two breakpoints of the Alu element insertion.

Analysis of long-read sequencing data revealed two *de novo* retrotransposon insertions in two samples. In the TSC-T20 sample, a *de novo* Alu element insertion, c.4006-18_4006-17insAlu4006-17_4006-4dup, was identified within intron 31 of *TSC2*. The Alu element was inserted in the opposite orientation and showed size variations in the reads, ranging from 339 bp to 350 bp (Figure 4A). This Alu element was found to have a ∼50-nucleotide poly-A tail (the exact number of A residues could not be determined) followed by a 14-bp duplication of the pre-insertion wild-type sequence. The Alu element sequence was determined to be the most similar to the AluY element, with 2.5% divergence to the respective consensus sequence according to RepeatMasker (https://www.repeatmasker.org/). To confirm the insertion, primers were designed to amplify the inserted fragment and a further sequencing primer was used to sequence the two breakpoints (Figures 4B–D).

**FIGURE 5:**
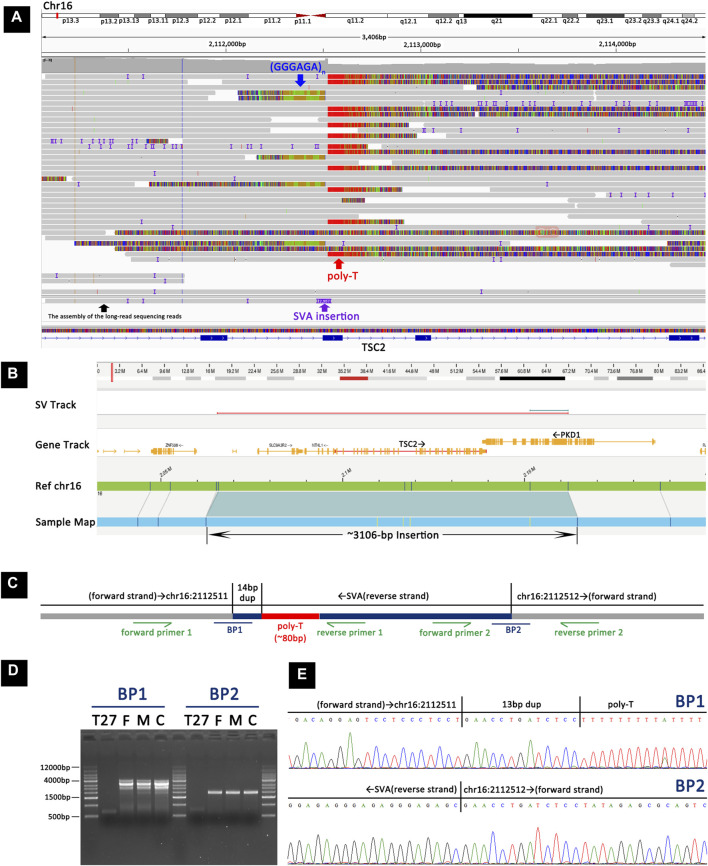
Detection and validation of the SVA element insertion in TSC-T27 by long-read sequencing, OGM, and Sanger sequencing. **(A)** Long-read sequencing revealed a heterozygous SVA element insertion within exon 12 of *TSC2*. **(B)** OGM showing a heterozygous ∼3106-bp insertion in *TSC2*. **(C)** A schematic representation of the SVA element insertion. BP1 and BP2 represent the two breakpoint sequences. Primers were designed to amplify and sequence the two breakpoint junction sequences. **(D)** Agarose gel electrophoresis of PCR products indicated that the insertion was present in TSC-T27 but not in her parents or the control sample. **(E)** Sanger sequencing of the PCR products from TSC-T27 validated the two breakpoint junctions of the SVA element insertion.

In TSC-T27, a *de novo* exonic SINE-VNTR-Alus (SVA) insertion was identified within exon 12 of *TSC2* (Figure 5). This SVA insertion was in the antisense orientation and was accompanied by a 13-bp target site duplication and a poly-A tail of ∼80 bp. The assembly of the long-read sequencing reads suggested that the SVA element was ∼2,957 bp in size. The OGM platform suggested a heterozygous ∼3,106-bp insertion in *TSC2*; however, according to the principle of OGM, the size provide by OGM could have a margin of error of several hundred base pairs relative to the actual size, and thus, the OGM results are in agreement with the results of long-read sequencing. To further confirm the insertion, two primers were designed to amplify and sequence the two breakpoint junction products (Figures 5B–E). By examining 12 short tandem repeats (STRs), the biological relationship between the parents and the patient was confirmed.

### Small insertions and deletions

Patients with NMI revealed three small insertion–deletion variants (InDels), comprising germline insertions, a mosaic small InDel, and a mosaic small deletion. TSC-T11 was found to have a *de novo* heterozygous 24-bp duplication (c.4937_4960dup) on *TSC2* (Supplementary Figure S7), and the biological relationship between the parents and TSC-T11 was confirmed by examining 12 STRs. TSC-T17 was identified to have a mosaic InDel at VAF 9.7%, which involved the deletion of 13-bp nucleotides from and insertion of 26-bp nucleotides in exon 37 of *TSC2* (Supplementary Figure S8). TSC-T28 was observed to have a mosaic 454-bp deletion at VAF 6%, involving the majority of exon 12 and a part of intron 12 (Supplementary Figure S9). All variants were then verified through PCR and sequencing.

### Germline splice variation

Two unrelated patients, TSC-T3 and TSC-T4, were found to have a heterozygous splice site variation on *TSC2*: c.848 + 281C>T (Supplementary Figure S10). This variant has been reported multiple times in patients with tuberous sclerosis (Mayer et al., 1999; Tyburczy et al., 2015; Bertoli-Avella et al., 2021), and research has revealed that it activates a splice donor site, leading to the insertion of an 89-bp pseudoexon and the introduction of a premature termination codon (Mayer et al., 1999; Mayer et al., 2000), thus classifying it as pathogenic. Upon communication with the company that performed deep sequencing for these two patients, it was discovered that their products did not cover the region in which the variant was located, which was the primary cause of the disease-causing variant of these two patients being missed.

### Further evaluate the effectiveness of targeted long-read sequencing

To further evaluate the efficacy of targeted long-read sequencing in detecting various types of genetic variations, three individuals (TSC-C1, TSC-C2, and TSC-C3) harboring variations that were otherwise not seen in the NMI cohort were chosen. TSC-C1 had a large deletion of the entire *TSC1*, which was revealed by MLPA. The depth of *TSC1* in TSC-C1 was found to be significantly decreased upon long-read sequencing, and there was a lack of heterozygosity across the entire *TSC1*, suggesting the deletion of *TSC1*. Meanwhile, TSC-C2 and TSC-C3 had small InDels, a common variation type in patients with TSC. Notably, 20 and 30 reads were identified upon long-read sequencing, which supported the presence of InDels, respectively. All three variants were accurately identified by targeted long-read sequencing, demonstrating the detection capability of long-read sequencing.

### Genotype–phenotype correlations

In this study, 26 tuberous sclerosis patients with NMI were recruited, all of whom were clinically diagnosed with definite tuberous sclerosis and presented with specific clinical symptoms outlined in Supplementary Table S3. Among these 26 patients, TSC-T28 was a fetus diagnosed with tuberous sclerosis following induced abortion, whereas the age of the remaining 25 patients (12 females and 13 males) ranged from 2 to 39 years. Among these 25 patients, 16 had seizures, with the age of onset ranging from 4 months to 10 years; these 16 individuals included 10 individuals with drug-resistant epilepsy.

**FIGURE 6:**
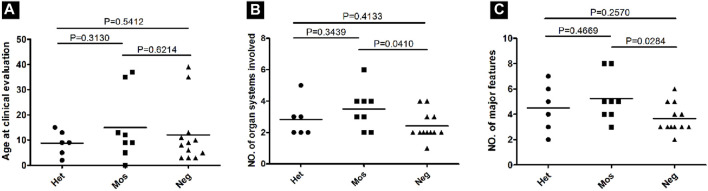
Correlation between the phenotype and the variant status in 26 patients with NMI. **(A)** Age at the time of clinical evaluation was compared among patients with various variant statuses, including heterozygous (Het), mosaic (Mos), and negative (Neg). *P* values were determined using a t-test. **(B)** The number of organ systems involved for each patient sorted according to the variant status. **(C)** The number of major features involved for each patient sorted according to the variant status.

To assess the clinical phenotype of patients with different pathogenic variant detection statuses (heterozygous, mosaic, and negative), we evaluated the major and minor features of the cases based on the latest diagnostic criteria for tuberous sclerosis. We considered the number of organs affected (the skin, brain, eye, heart, kidney, and lung) and the number of major features. Within our cohort, there were no phenotypic biological differences observed between men and women. We examined the correlation between the variant status and age as the number of symptoms may increase with age. Nevertheless, no significant differences in age at the time of clinical evaluation were noted among heterozygous, mosaic, and negative groups, implying that a difference in age did not explain the correlation between disease severity and pathogenic variant detection status (Figure 6A).

The only case involving six organ systems had a mosaic large deletion (VAF = 14.3%), which was 37 kb in size and encompassed exons 1–3 of *NTHL1* and exons 1–25 of *TSC2*, along with a partial exon 26. The average number of organ systems affected was 2.8 for heterozygous variant cases, 3.67 for mosaic variant cases, and 2.57 for cases with negative results. Although the trend showed that more organ systems were affected in mosaic patients, the differences among the three groups were not statistically significant (t-test, two-tailed, *p* > 0.05; Figure 6B). Similarly, the average number of major features was 4.2 for heterozygous variant cases, 5.67 for mosaic variant cases, and 3.67 for cases with negative results, with the differences between negative and mosaic variant cases being statistically significant (*p* = 0.0284; Figure 6C).

## Discussion

Herein, we report on our analysis of 26 patients having TSC who were not identified as having a pathogenic variant through conventional molecular diagnostic analyses of *TSC1* and *TSC2*. We were able to identify the variant with a high suspicion of pathogenicity in 14 cases (53.8%). Of these 14 patients, 8 patients were found to have SVs: four had InDels, two had splice variants. Notably, the majority (57.1%) of the identified pathogenic variants (8/14, 57.1%) were mosaic variants, which is consistent with previous studies of patients having tuberous sclerosis with NMI (Tyburczy et al., 2015; Giannikou et al., 2019; Ye et al., 2022; Klonowska et al., 2023). Ye et al. (Ye et al., 2022) and Tyburczy et al. (Tyburczy et al., 2015) reported in their studies that 5.5% and 7.5% of patients with tuberous sclerosis had mosaic pathogenic variants, respectively. Our study provides further evidence that mosaicism occurs in a higher proportion of TSC patients than expected.

In recent years, research involving patients having tuberous sclerosis with NMI has mainly been devoted to taking advantage of deep sequencing, leading to the identification of numerous mosaic and deep intronic variants (Mayer et al., 1999; Weren et al., 2015; Ye et al., 2022; Klonowska et al., 2023); however, the pathogenic variants of some patients remained undiscovered. To address this, we chose long-read sequencing in our study to identify different pathogenic variants. Moreover, half of the cases we included had already undergone deep sequencing. Consequently, the variants we found were mainly SVs, such as mosaic large deletions, complex SVs, and retrotransposon insertions, which are hard to detect using short-read sequencing. Our findings indicated the presence of various pathogenic variants in patients having tuberous sclerosis with NMI that would be overlooked in clinical genetic testing, such as mosaic small variants, mosaic structure variants, deep intron variants, retrotransposon insertions, and complex structural variations. Furthermore, it was proposed that mosaic structural variations and special variant types like Alu element insertions are challenging to identify using second-generation sequencing and MLPA, and long-read sequencing may be a potential choice for cases of tuberous sclerosis with NMI.

We used a combination of molecular analytic tools, including targeted long-read sequencing, Sanger sequencing, OGM, and ONT-WGS, to accurately detect a mosaic complex structural variant that spanned ∼83 kb in TSC-T5. Targeted long-read sequencing revealed the complex structural variation, whereas OGM, with its high read length of 250 kb, allowed for the conformation of the true size of the variant. With an average read length of 50 kb, although ONT-WGS could not show the full picture of the entire variant in one read, but it could provide more information about the breakpoint junction sequences than targeted long-read sequencing, which allowed us to identify the variant. Finally, Sanger sequencing allowed us to verify the authenticity of the connections and the insertion of few bases in the connection fragments. This approach successfully presented the variant and provided a useful strategy for verifying other structural variations in the future. SVs arise from various mutational processes, such as DNA recombination-, replication- and repair-related processes (Carvalho and Lupski, 2016). The structural variant in TSC-T5 is novel and nonrecurrent. Its underlying mechanism, however, still needs to be confirmed.

We discovered two different retrotransposon insertions in our cases, including an intronic Alu element in TSC-T20 and an exonic SVA insertion in TSC-T27. To our knowledge, this is the first report of retrotransposon insertions in *TSC2* in the context of tuberous sclerosis. Alu element and SVA belong to non-LTR retrotransposons, and there is clear evidence that non-LTR retrotransposons are still active in humans, as demonstrated in the dozens of reported cases of *de novo* insertions that are associated with genetic disorders (Wallace et al., 1991; Oldridge et al., 1999; Claverie-Martin et al., 2003; Cordaux and Batzer, 2009; Ade and Roy-Engel, 2016). Alu elements, which are primate-specific retrotransposons, were formed by the 50-to-30 fusion of the 7SL RNA gene and have been present in the human genome for over 65 million years, with the total number of copies surpassing one million (Hasler and Strub, 2006; Kriegs et al., 2007). *De novo* Alu insertions close to the splice acceptor and splice donor sites can reportedly disrupt splicing, resulting in disease-causing alleles (Wallace et al., 1991; Tighe et al., 2002; Gallus et al., 2010; Payer et al., 2019). Moreover, Alu elements that are antisense to a gene can provide splice acceptor sites (Sorek et al., 2004; Krull et al., 2005). The RNA Splicer developed by the Research Institute of Tsinghua predicted that the Alu element insertion of TSC-T20 would lead to an 82-bp pseudo-exon insertion in the RNA. We assume that the pathology caused by this Alu element insertion is due to the disruption of splicing. SVAs are the youngest retrotransposon family in the human genome (Pfaff et al., 2022) and typically measure ∼2 kb in length with ∼3,000 copies present (Cordaux and Batzer, 2009). The full-length SVA identified in TSC-T27 is 3 kb in length and located in exon 12 of *TSC2*. Thus far, more than 10 cases of exonic SVA insertions have been linked to Mendelian diseases (Pfaff et al., 2022). These insertions can lead to pathogenic conditions in two main ways. First, SVAs contain stop codons which can introduce a premature stop codon or cause a frameshift mutation when inserted into exons (Nakamura et al., 2015; Delvallee et al., 2021; Yamamoto et al., 2021; Hiraide et al., 2022). Second, SVA insertion may interfere with the normal splicing of the gene, thus disrupting its normal function (Nakamura et al., 2015; Jones et al., 2020; Yamamoto et al., 2021).

This study has several potential limitations. First, we studied a relatively small number of patients and made phenotype comparisons among them based on their pathogenic variant detection status (heterozygous, mosaic, and negative). Although the average number of major features with statistically significant differences between negative and mosaic variant cases is notable, it is essential to consider the limited sample size. Second, to ensure a reliable confirmation of mosaicism in a patient, the use of two different methodologies is recommended. We used various techniques, including Sanger sequencing, OGM, long-read whole-genome sequencing by Oxford Nanopore technology, ddPCR, and MLPA, to verify the mosaic variants identified; however, except for ddPCR, none of these techniques can accurately ascertain the VAF of the mosaic variants, and thus, the VAF should be interpreted with caution. Third, even though we used the RNA Splicer (https://rddc.tsinghua-gd.org/ai/rna-splicer) to predict the effect of Alu element insertion on RNA which occurred *de novo*, we could not acquire patient sample for RNA analysis to corroborate our predictions. Fourth, 8 cases remain unsolved, possibly due to the following limitations of our study. 1) Mosaic variants are limited to the affected organs (kidney and brain) and not detectable in peripheral blood samples used in this study (Klonowska et al., 2023). 2) Mosaic SVs that have at least one breakpoint in *TSC2* or *TSC1* with a VAF lower than 2% and small variants with a VAF below 5% were below the limit of detection using our methods. 3) mosaic SVs that encompass the entire *TSC1* or *TSC2* may have been missed by our methods because the junction sequence could not be captured by the probes.

In our research, we used long-read sequencing in patients with tuberous sclerosis in whom a pathogenic or likely pathogenic variant of *TSC1* or *TSC2* could not be identified using short-read sequencing or other traditional methods, and we have been able to detect a pathogenic variation in *TSC1* or *TSC2* in the majority of these patients using long-read sequencing. Our findings broaden the range of pathogenic variants associated with tuberous sclerosis, particularly in terms of complex SVs and retrotransposon insertions that have never been documented in tuberous sclerosis patients. It appears much more likely that rather than having an additional gene causing typical tuberous sclerosis, individuals who have persistently had NMI have either a mosaic variation that was not present in blood or an extremely mosaic variant, which our methods could not reveal. Owing to the current high costs and limited availability of long-read sequencing, we recommend next-generation sequencing as the primary genetic testing method for individuals with tuberous sclerosis. However, given the ability of long-read sequencing to uncover mosaic large deletions, complex structural variations, retrotransposon insertions, and other typical variants, it may be a suitable approach for patients having tuberous sclerosis with NMI, particularly when patients seek to identify the pathogenic variation for reproductive planning. Additionally, our findings are applicable to various other genetic disorders, thus emphasizing the importance of using long-read sequencing to identify variants in unresolved cases wherein a causative variant could not be identified.

## Data Availability

All variants have been submitted to the LOVD database at the following links: https://databases.lovd.nl/shared/genes/TSC1 and https://databases.lovd.nl/shared/genes/TSC2. The assigned identification numbers range from 00451722 to 00451734.

## Abbreviations

bp: base pair; ddPCR: droplet digital PCR; MLPA: multiplex ligation-dependent probe analysis; NMI: no mutation identified; NGS: next-generation sequencing; InDels: insertion–deletion variants; OGM: optical genome mapping; SV: structural variant; SNVs: single nucleotide variants.
