# Supplementary material for: Uncovering hidden genetic variations: long-read sequencing reveals new insights into tuberous sclerosis complex
Source: Front Cell Dev Biol. 2024 Jul 31;12:1415258. doi: 10.3389/fcell.2024.1415258 (PMC11321964; doi:10.3389/fcell.2024.1415258)
Supplement: Supplementary file 1 [file DataSheet1.docx]

Supplementary material

Supplemental Table S**1. Primers and probes for droplet digital PCR**

| Name | Primer/Probe Sequence**(5′-3′)** | Region of interest (hg19) |
| --- | --- | --- |
| TSC2-E19-F | CAGAGAGAGGCTCTGAGAAG | chr16:2121790—2121909 |
| TSC2-E19-R | GGAAGAGCAGGGAGTAGG |  |
| TSC2-E19-VIC | HEX-TG+G+CC+C+GGCG-3IABkFQ* |  |
| TSC2-E21-F | CTCCAGAACTGACTTGCACCTG | chr16:2122900—2122978 |
| TSC2-E21-R | GGTTTTGTCCAGGTAGTTATGGTAA |  |
| TSC2-E21-VIC | HEX-CCAGTGCTGACAGCAT-MGB |  |
| RPP30-F | TGAAGAAACCTCGGCCATC | chr10:92660363-92660432 |
| RPP30-R | CCCTCACACTTGGCTTTCT |  |
| RPP30-FAM | FAM-AGATGAAGATTGTCTTCCAG-MGB |  |

*plus sign “+” represents locked nucleic acid (LNA)

Supplemental Table S2. ACMG interpretation of variants

| Patient | Gene | Variant | Variation type | Source of variation | ACMG  Pathogenicity | | ACMG criteria |
| --- | --- | --- | --- | --- | --- | --- | --- |
| Germline variations | | | | | |  |  |
| TSC-T3 | *TSC2* | c.848+281C>T | Splice | *de novo^#^* | Pathogenic | | PVS1, PS2, PM2_supporting, PP4 |
| TSC-T4 | *TSC2* | c.848+281C>T | Splice | Not maternal | Pathogenic | | PVS1, PS2, PM2_supporting, PP4 |
| TSC-T20 | *TSC2* | c.4006-18_4006-17insAlu4006-17-4006-4dup | InDel | *de novo** | VUS | | PM2_supporting, PP4, PP3 |
| TSC-T11 | *TSC2* | c.4937_4960dup | InDel | *de novo^#^* | Likely pathogenic | | PS2, PM4, PM2_supporting, PP4 |
| TSC-T25 | *TSC2* | NC_000016.9:2091899-2130541del | SV | NA | Pathogenic | | PVS1, PM2_supporting, PP4 |
| TSC-T27 | *TSC2* | NM_000548.3:c.1271_1272insSVA1272_1285dup | SV | *de novo^#^* | Likely Pathogenic | | PS2, PM4, PM2_supporting, PP4 |
| Mosaic variations | |  |  | | |  |  |
| TSC-T5 | *TSC2* | NC_000016.9:g.2117813_2117814ins[GG;2086537_2115686inv;GCTTGCAGGTGCGCAT;2115777_2127813dup] | SV | / | Pathogenic | | PVS1, PS2, PM2_supporting, PP4 |
| TSC-T17 | *TSC2* | c.4715_4727delinsTGGCTCCTACAGGTACAGGTACAGAT NP_000539.2:p.(T1572Mfs*35) | InDel | / | Pathogenic | | PVS1, PS2, PM2_supporting, PP4 |
| TSC-T7 | *TSC1* | NC_000016.9:g.135782986-135789396del | SV | / | Pathogenic | | PVS1, PS2, PM2_supporting, PP4 |
| TSC-T12 | *TSC2* | c.2085_2546-384del  NC_000016.9:2121923-2125416del | SV | / | Pathogenic | | PVS1, PS2, PM2_supporting, PP4 |
| TSC-T15 | *TSC2* | NC_000016.9:2093996-2127706del | SV | / | Pathogenic | | PVS1, PS2, PM2_supporting, PP4 |
| TSC-T16 | *TSC2* | NC_000016.9:2118894-2125546del | SV | / | Pathogenic | | PVS1, PS2, PM2_supporting, PP4 |
| TSC-T23 | *TSC2* | NC_000016.9:2127219-2142640del | SV | / | Pathogenic | | PVS1, PS2, PM2_supporting, PP4 |
| TSC-T28 | *TSC2* | NM_000548.3:c.1130_1258-163del | InDel | / | Pathogenic | | PVS1, PS2, PM2_supporting, PP4 |

*^#^***:** *de novo with confirmed parental relationships;* ***:** *de novo with unconfirmed parental relationships.*

Abbreviations: ACMG, American College of Medical Genetics guideline; SV: structural variant; InDels: insertion–deletion variants; VUS: variant of uncertain significance; NA: unavailable
Supplemental Table S3 Phenotype of the included tuberous sclerosis patients

| Patient | Age  (years) | Sex | Major features | | | | | | | | | | |  |
| --- | --- | --- | --- | --- | --- | --- | --- | --- | --- | --- | --- | --- | --- | --- |
|  |  |  | Hypomelanotic macules (≥3) | Angiofibroma (≥3) or fibrous cephalic plaque | Ungual fibromas (≥2) | Shagreen patch | Multiple retinal hamartomas | Multiple cortical tubers and/or radial migration lines | Subependymal nodule (≥2) | Subependymal giant cell astrocytoma | Cardiac rhabdomyoma | LAM | Angiomyolipomas (≥2) | Minor features |
| TSC-T1 | 11 | F | - | - | - | + | - | + | + | + | - | NA | - | Nonrenal hamartomas |
| TSC-T2 | 6 | F | + | + | - | - | NA | + | - | - | - | NA | - | - |
| TSC-T3 | 5 | F | + | - | - | - | NA | + | + | - | Mutiple | NA | - | - |
| TSC-T4 | 9 | F | + | - | - | - | + | + | + | + | Mutiple | NA | + | Nonrenal hamartomas |
| TSC-T5 | 13 | F | - | + | - | - | - | + | + | - | - | NA | + | - |
| TSC-T6 | 3 | M | - | - | - | + | NA | + | + | + | - | NA | - | - |
| TSC-T7 | 35 | F | + | + | - | + | NA | NA | NA | NA | - | + | + | - |
| TSC-T8 | 3 | M | - | - | - | - | NA | + | + | - | - | - | - | - |
| TSC-T9 | 3 | M | + | - | - | - | NA | + | + | - | - | NA | - | Multiple renal cysts |
| TSC-T10 | 8 | F | - | - | - | - | - | + | + | - | Mutiple | NA | - | - |
| TSC-T11 | 13 | M | + | - | - | - | NA | + | - | - | - | NA | NA | NA |
| TSC-T12 | 9 | M | - | - | - | + | + | + | + | - | - | NA | + | - |
| TSC-T13 | 10 | M | + | + | - | + | - | + | + | - | - | + | - | - |
| TSC-T14 | 5 | M | - | - | - | - | - | + | + | - | - | NA | + | - |
| TSC-T15 | 9 | F | + | - | - | + | + | + | + | - | Mutiple | + | + | Multiple renal cysts, nonrenal hamartomas |
| TSC-T16 | 12 | M | + | - | - | + | + | + | - | - | - | - | + | - |
| TSC-T17 | 37 | F | + | + | + | - | NA | + | + | + | - | + | + | Nonrenal hamartomas |
| TSC-T18 | 39 | F | - | - | - | - | NA | + | - | - | - | + | + | NA |
| TSC-T19 | 35 | F | - | + | + | - | NA | + | - | - | - | + | + | Nonrenal hamartomas |
| TSC-T20 | 2 | M | + | - | - | - | - | + | - | + | - | - | - | - |
| TSC-T21 | 13 | F | - | + | - | - | - | + | + | - | - | - | - | - |
| TSC-T23 | 5 | F | + | - | - | + | - | + | + | - | - | - | - | Multiple renal cysts |
| TSC-T24 | 9 | F | + | - | - | - | - | + | + | - | Mutiple | - | + | Multiple renal cysts |
| TSC-T25 | 15 | F | + | + | - | + | - | + | + | - | - | - | - | Nonrenal hamartomas |
| TSC-T27 | 9 | F | + | + | - | + | - | + | + | - | Mutiple | - | - | - |
| TSC-T28 | / | NA | NA | NA | NA | NA | NA | + | + | - | Unique | - | - | - |

Supplemental Table S4 Primers used in paternity testing by STR analysis

| **Primer ID** | **Primer Sequence (5′-3′)** | **Label** |
| --- | --- | --- |
| D6S1043-FAM-110 | CAAGGATGGGTGGATCAATA | FAM |
| D6S1043-R-110 | TTGTATGAGCCACTTCCCAT |  |
| D13S317-HEX-185 | ACAGAAGTCTGGGATGTGGA | HEX |
| D13S317-R-185 | GCCCAAAAAGACAGACAGAA |  |
| D16S539-ROX-224 | CCCATTTTTATATGGGAGCAAA | ROX |
| D16S539-R-224 | AACAGCCTACAGAGTGATTCCA |  |
| D2S441-FAM-397 | TCCTGAACCCAGTCCTCTTG | FAM |
| D2S441-R-397 | TTCACTCTCCTTCCCAAATGTT |  |
| AMEL-HEX-85 | CCTGGGCTCTGTAAAGAATAGT | HEX |
| AMEL-R-85 | GCTGGTGGTAGGAACTGTAAAAT |  |
| TPOX-FAM-124 | CACTAGCACCCAGAACCGTC | FAM |
| TPOX-R-124 | CCTTGTCAGCGTTTATTTGCC |  |
| D12S391-HEX-225 | AACAGGATCAATGGATGCAT | HEX |
| D12S391-R-225 | TGGCTTTTAGACCTGGACTG |  |
| FGA-ROX-271 | TCACGGTCTGAAATCGAAAA | ROX |
| FGA-R-271 | GCAGGGCATAACATTATCCAA |  |
| D3S1358-FAM-330 | GCCATATTCACTTGCCCACT | FAM |
| D3S1358-R-330 | AAAATTAGCCGGACATGGTG |  |
| Vwa-FAM-151 | GCCCTAGTGGATGATAAGAATAATCAGTATGTG | FAM |
| Vwa-R-151 | GGACAGATGATAAATACATAGGATGGATGG |  |
| TH01-HEX-161 | GTGGGCTGAAAAGCTCCCGATTAT | HEX |
| TH01-R-161 | GTGATTCCCATTGGCCTGTTCCTC |  |
| D18S51-ROX-305 | GAGCCATGTTCATGCCACTG | ROX |
| D18S51-R-305 | CAAACCCGACTACCAGCAAC |  |
| D2S1338-FAM-353 | TCCTACCAGAATGCCAGTCC | FAM |
| D2S1338-R-353 | GTGGAGTGGAGGTGCCTAAA |  |


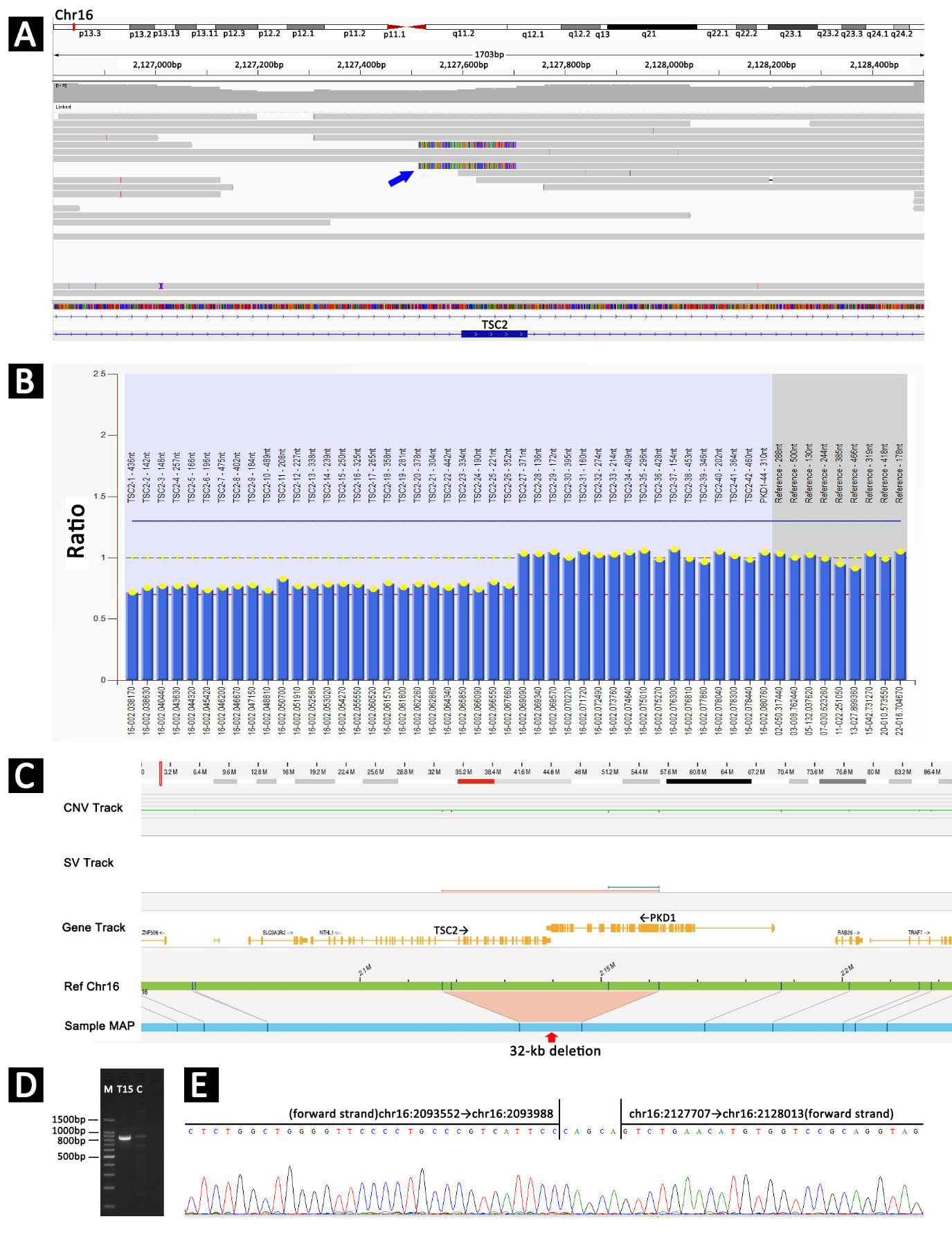


**Figure S1.** Detection and validation of the mosaic large deletion in TSC-T15 via long-read sequencing, MLPA, OGM, and Sanger sequencing. (A) Long–read sequencing revealed that two reads indicated mosaic deletions in the chr16:2093955-2127706 region. (B) MLPA analysis of *TSC2* revealed that the ratio of probes in exons 1–24 was slightly lower than that in the normal region (0.8–1.2) but higher than that in the heterozygous deletion (0.40–0.65), suggesting an ambiguous copy number. (C) OGM shows a 32-kb deletion overlapping *TSC2* and an insertion of ~2.3 kb in *PKD1*. This insertion variant was homozygous, which caused the size and location of the deletion to be slightly smaller and closer to *PKD1* than what was indicated by long-read sequencing results. (D) Agarose gel electrophoresis of PCR products of breakpoint junction of the deletion from TSC-T15 and a healthy control. (E) Sanger sequencing of the PCR products from TSC-T15 validated the break junction of the deletion and the base mapped to hg19.


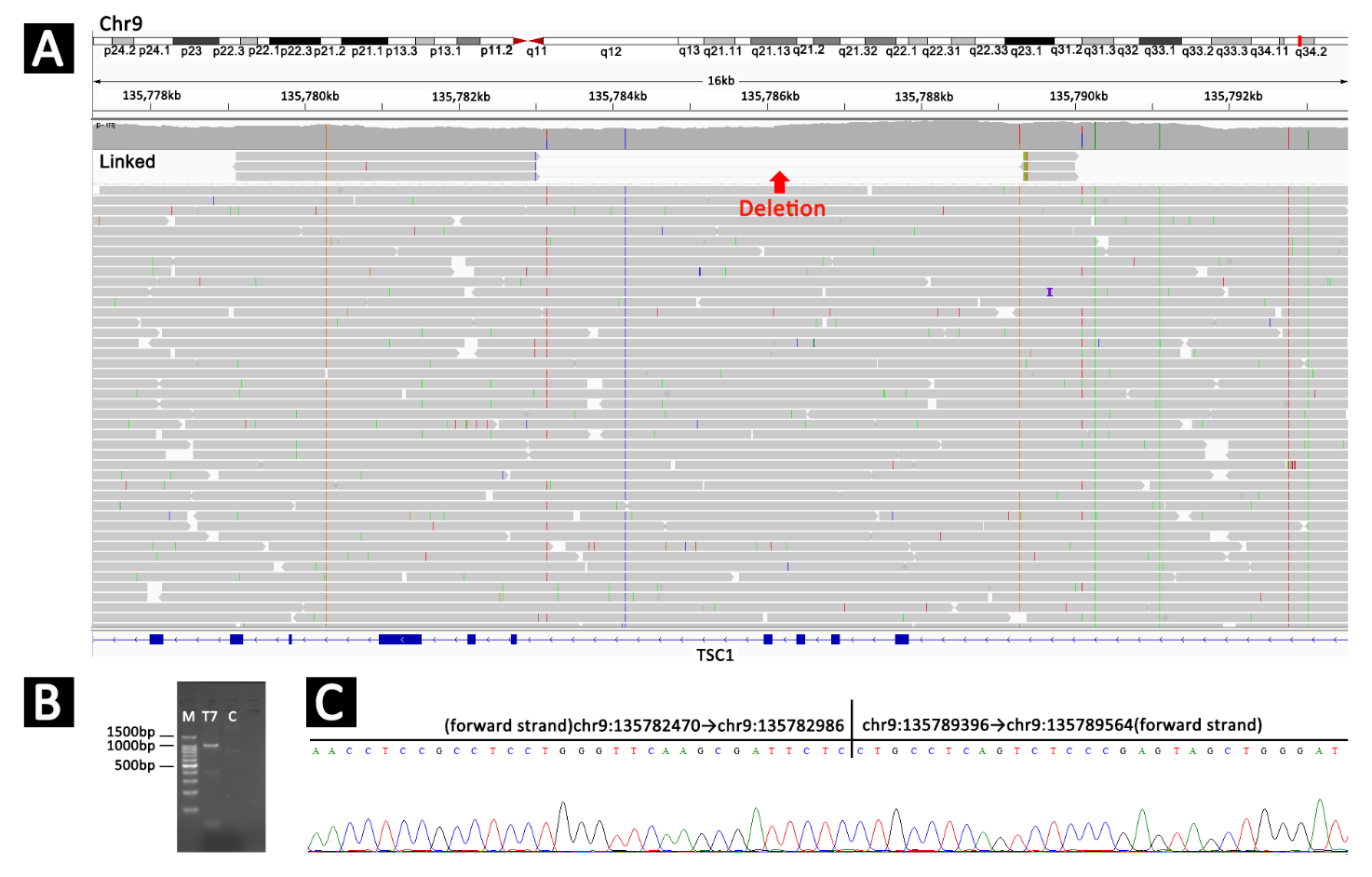


**Figure S2.** Detection and validation of the mosaic mutation in TSC-T7 by long-read sequencing and Sanger sequencing. (A) Long-read sequencing revealed mosaic large deletion in *TSC1*. (B) Agarose gel electrophoresis of PCR products of breakpoint junctions of the deletion from TSC-T7 and a healthy control. (C) Sanger sequencing of the PCR products from TSC-T7 validated the break junction of the deletion and base complementarity to hg19.


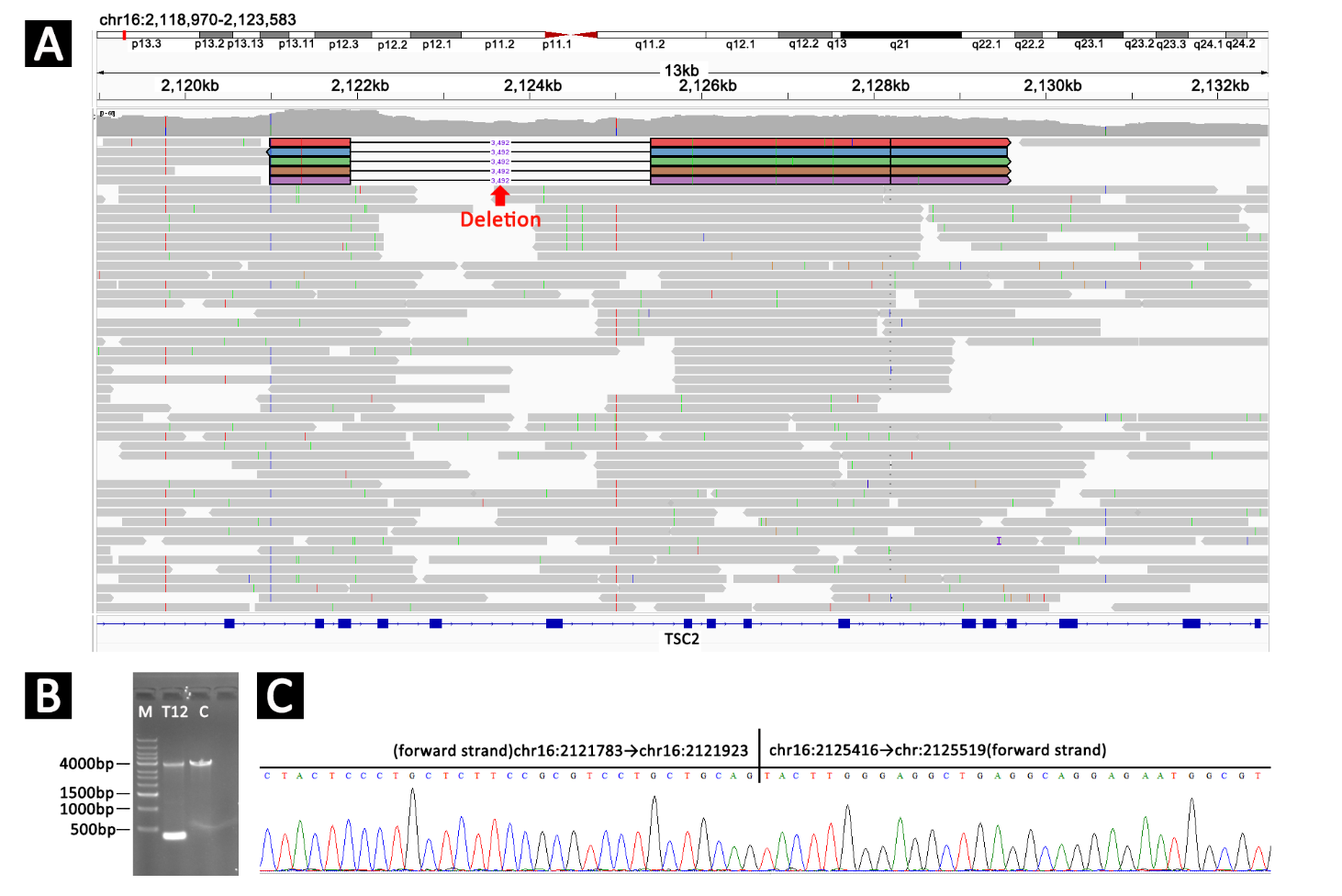


**Figure S3.** Detection and validation of the mosaic mutation in TSC-T12 by long-read sequencing and Sanger sequencing. (A) Long-read sequencing revealed mosaic large deletion in *TSC2*. (B) Agarose gel electrophoresis of PCR products of breakpoint junctions of the deletion from TSC-T12 and a healthy control. (C) Sanger sequencing of the PCR products from TSC-T12 validated the break junction of the deletion and base complementarity to hg19.


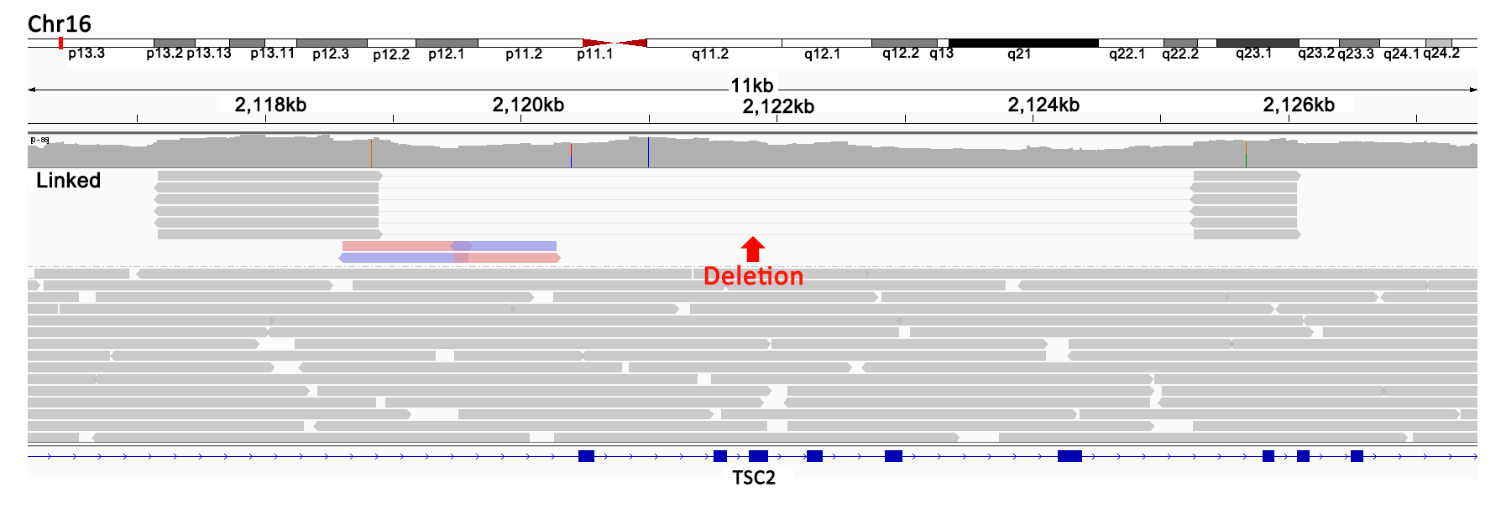


**Figure S4.** Long-read sequencing revealed mosaic large deletion (chr16:2118894-2125546del) in TSC-T16


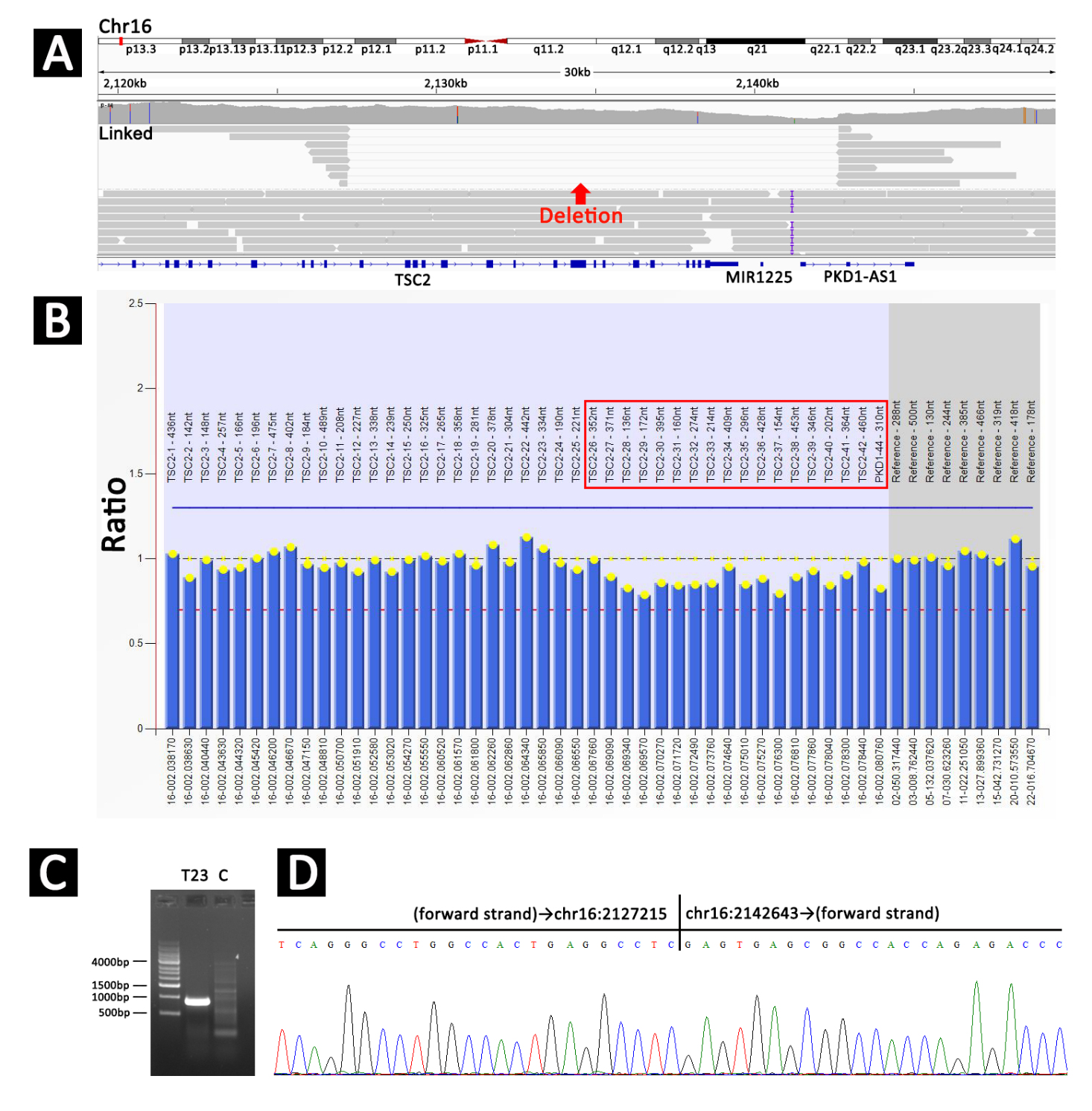


**Figure S5.** Detection and validation of the mosaic large deletion in TSC-T23 by long-read sequencing, MLPA, and Sanger sequencing. (A) Long-read sequencing revealed that eight reads indicated mosaic deletions in the chr16:2127219-2142640 region. (B) MLPA analysis revealed that the final ratio of probes in exons 26–42 of *TSC2* and exon 44 of *PKD1* was slightly lower than that in the normal region but was still within the normal range (0.80 < final ratio < 1.20). (C) Agarose gel electrophoresis of PCR products of breakpoint junctions of the deletion from TSC-T23 and a healthy control. (E) Sanger sequencing of the PCR products from TSC-T23 validated the break junction of the deletion and base complementarity to hg19.


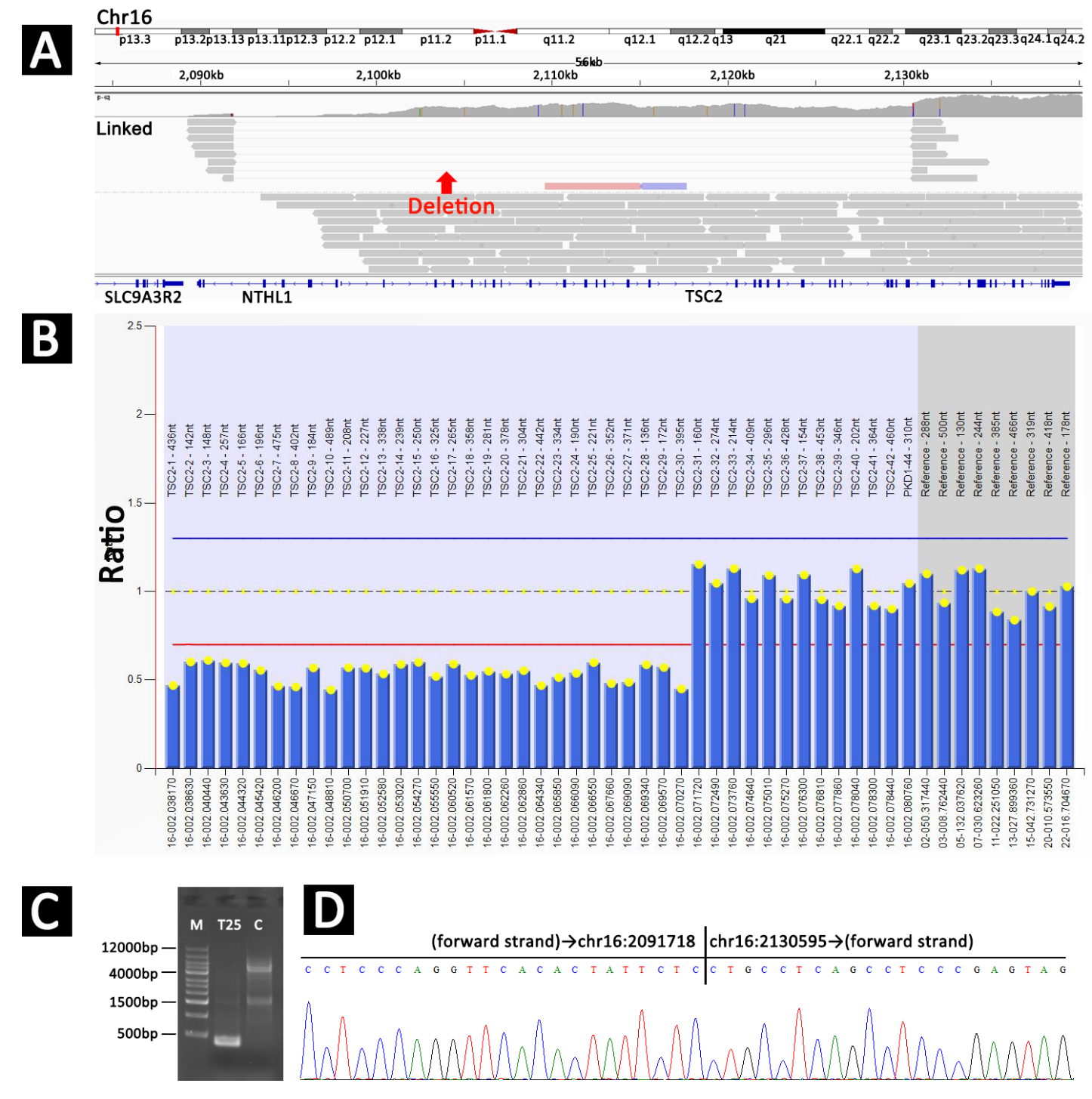


**Figure S6.** Detection and validation of the heterozygous large deletion in TSC-T25 by long-read sequencing, MLPA, and Sanger sequencing. (A) Long–read sequencing revealed deletions in the chr16:2091718-2130595 region. (B) MLPA analysis of the DNA of TSC-T25 revealed that exons 1–30 of *TSC2* were deleted. (C) Agarose gel electrophoresis of PCR products of breakpoint junctions of the deletion from TSC-T25 and a healthy control. (E) Sanger sequencing of the PCR products from TSC-T25 validated the break junction of the deletion and base complementarity to hg19.


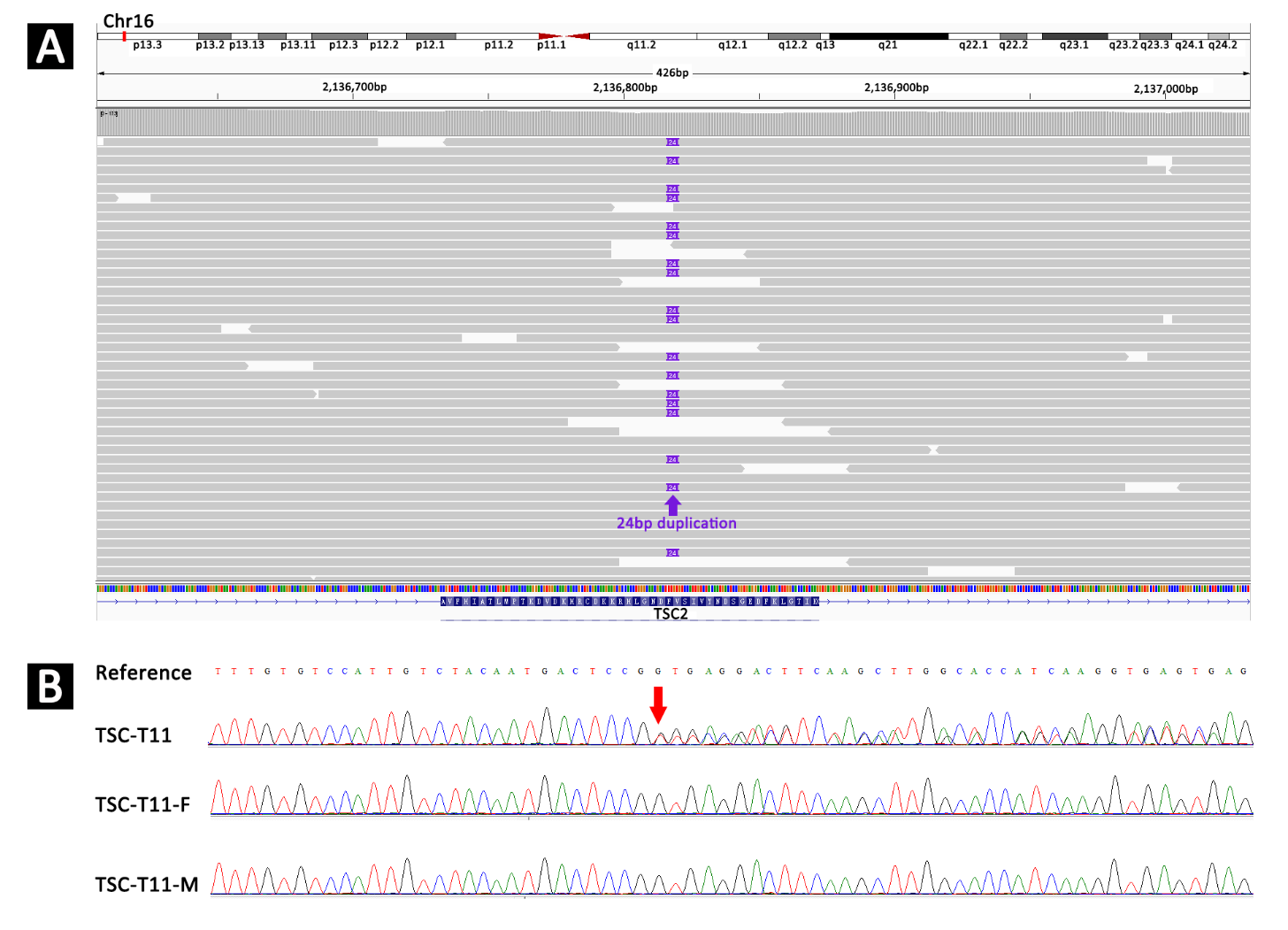


**Figure S7.** Detection and validation of the heterozygous mutation in TSC-T11 by long-read sequencing and Sanger sequencing. (A) Long-read sequencing revealed c.4937_4960dup in *TSC2*. (B) Sanger sequencing of TSC-T11 validated the mutation and base complementarity to hg19.


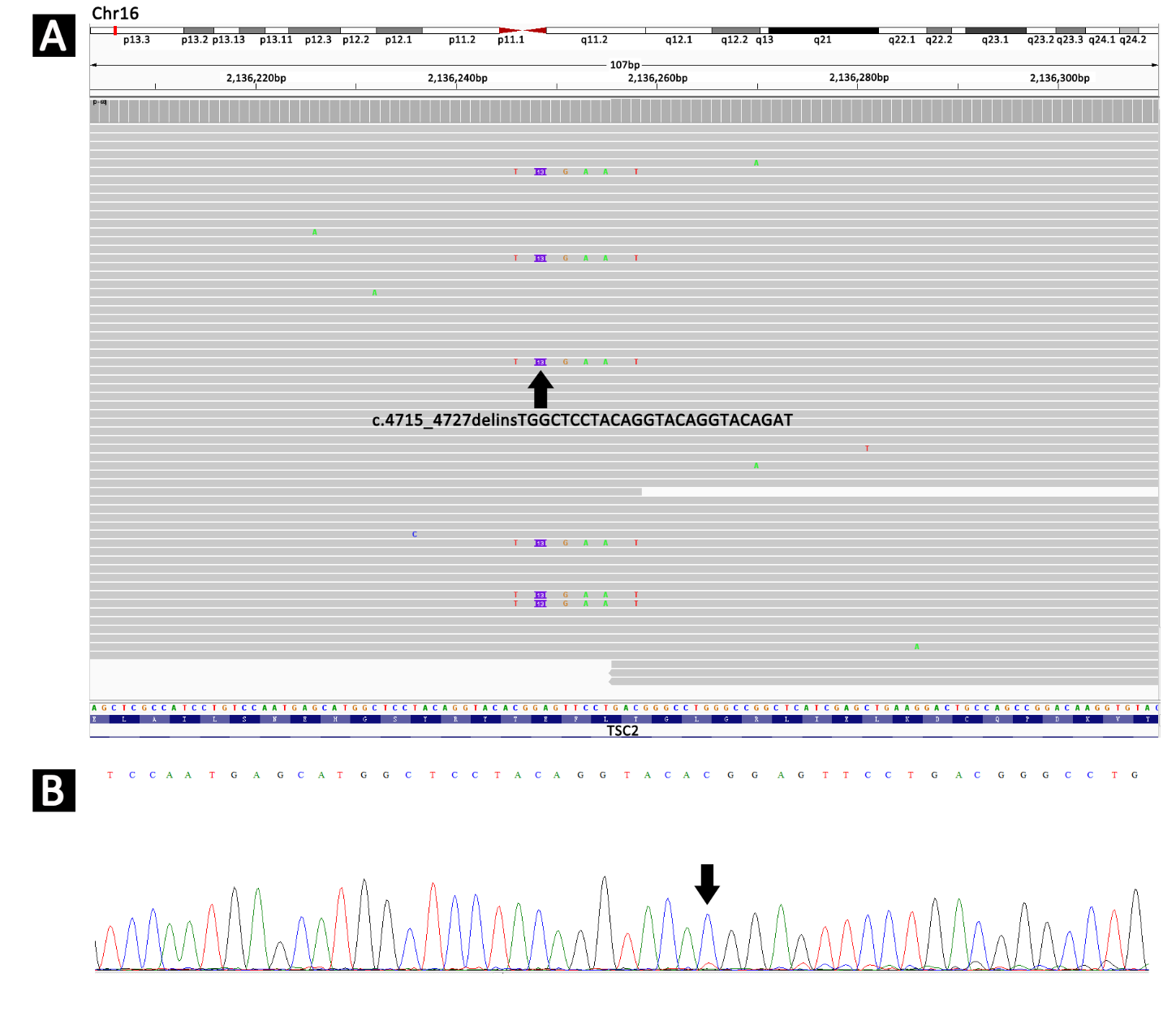


**Figure S8.** Detection and validation of the mosaic mutation in TSC-T17 by long-read sequencing and Sanger sequencing. (A) Long-read sequencing revealed mosaic c.4715_4727delinsTGGCTCCTACAGGTACAGGTACAGAT in *TSC2*. (B) Sanger sequencing of TSC-T17 validated the mutation and base complementarity to hg19.


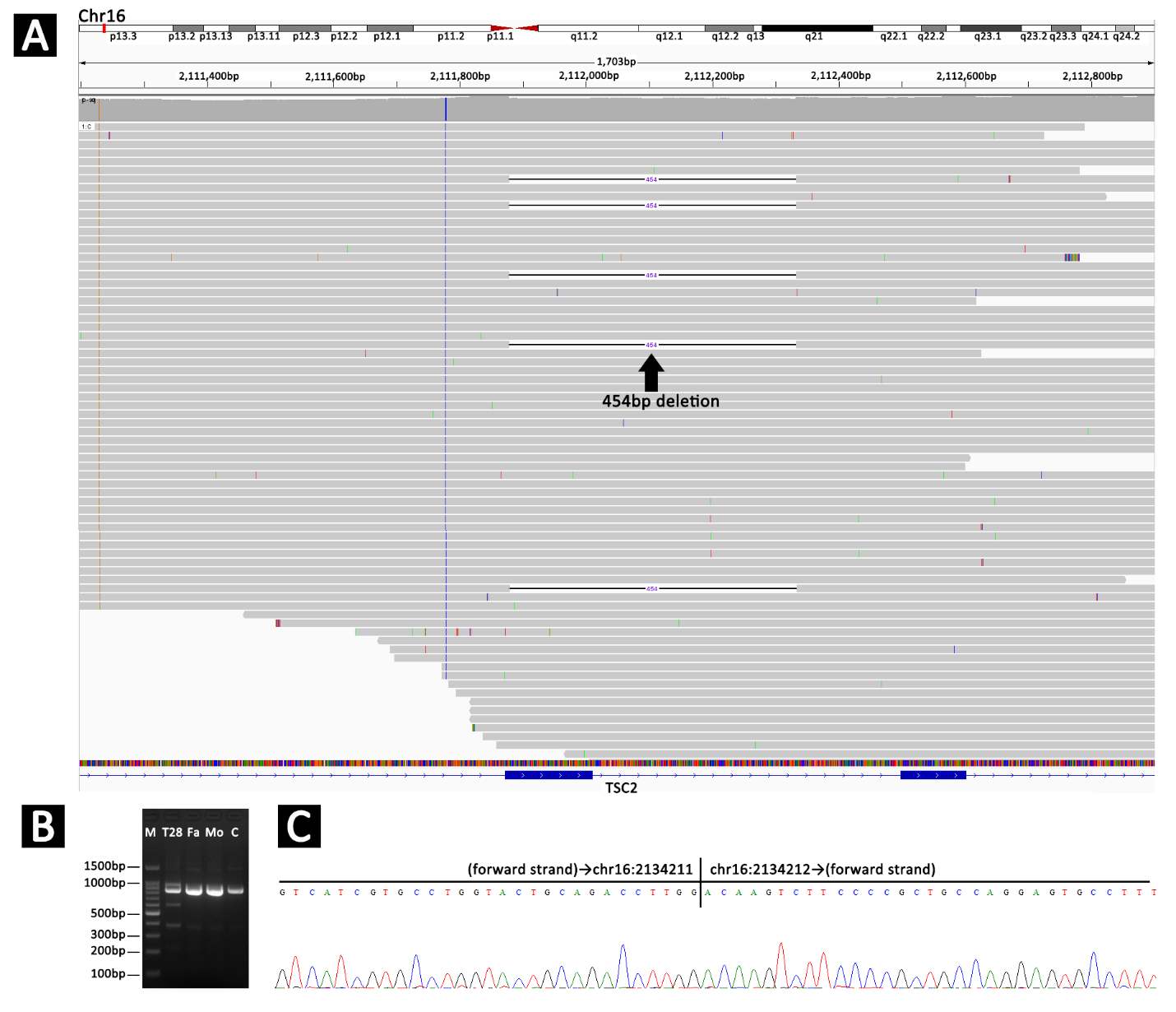


**Figure S9.** Detection and validation of the mosaic mutation in TSC-T28 via long-read sequencing and Sanger sequencing. (A) Long-read sequencing revealed mosaic c.1130_1258-163del in *TSC2*. (B) Agarose gel electrophoresis of PCR products of breakpoint junctions of the deletion from TSC-T28, her parents, and a healthy control. (C) Sanger sequencing of the PCR products from TSC-T28 validated the break junction of the deletion and base complementarity to hg19.


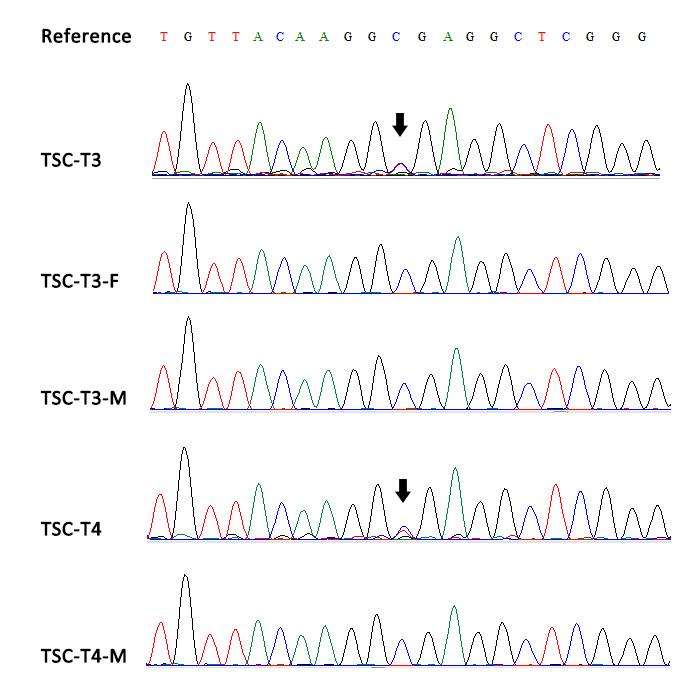


**Figure S10.** Sanger sequencing chromatograms of the c.848+281C>T variant in TSC-T3 and her parents and TSC-T4 and her mother.
